# Volume of Amygdala Subregions and Clinical Manifestations in Patients With First-Episode, Drug-Naïve Major Depression

**DOI:** 10.3389/fnhum.2021.780884

**Published:** 2022-01-03

**Authors:** Hirofumi Tesen, Keita Watanabe, Naomichi Okamoto, Atsuko Ikenouchi, Ryohei Igata, Yuki Konishi, Shingo Kakeda, Reiji Yoshimura

**Affiliations:** ^1^Department of Psychiatry, University of Occupational and Environmental Health, Kitakyushu, Japan; ^2^Open Innovation Institute, Kyoto University, Kyoto, Japan; ^3^Department of Radiology, Graduate School of Medicine, Hirosaki University, Hirosaki, Japan

**Keywords:** amygdala, subregions, major depression, MRI, first-episode, drug-naïve, hippocampus

## Abstract

We examined amygdala subregion volumes in patients with a first episode of major depression (MD) and in healthy subjects. Covariate-adjusted linear regression was performed to compare the MD and healthy groups, and adjustments for age, gender, and total estimated intracranial volume showed no differences in amygdala subregion volumes between the healthy and MD groups. Within the MD group, we examined the association between amygdala subregion volume and the 17-item Hamilton Rating Scale for Depression (HAMD) score and the HAMD subscale score, and found no association in the left amygdala. In the right amygdala, however, there was an inverse linear association between the HAMD total and the HAMD core and lateral nucleus and anterior-amygdaloid-regions. Furthermore, an inverse linear association was seen between the HAMD psychic and the lateral nucleus, anterior-amygdaloid-regions, transition, and whole amygdala. The findings of this study suggest that the severity of MD and some symptoms of MD are associated with right amygdala volume. There have been few reports on the relationship between MD and amygdala subregional volume, and further research is needed to accumulate more data for further validation.

## Introduction

Although the pathophysiology of major depression (MD) remains unclear, recent brain imaging studies using MRI have provided some interesting insights (Schmaal et al., 2016, 2017). For example, previous studies of the amygdala and hippocampus have reported reduced volume as a potential biomarker of depression (Roddy et al., 2019; Sheline et al., 2019). According to 12 studies reported so far, a 5.2% decrease in the left amygdala and a 7.4% decrease in the right amygdala have been reported in depression (Nolan et al., 2020). Notwithstanding, there are fewer reports on amygdala volume than on hippocampal volume, and there is a lack of assessment of the amygdala in relation to depression. One of the reasons for this is that the amygdala nuclei have been difficult to classify and identify. Additionally, the amygdala has different functions depending on its region (Maras and Petrulis, 2010; Alarcón et al., 2015). Therefore, it is meaningful to investigate the relationship between the amygdala subregional volume and clinical symptoms of depression, and the change in the amygdala subregional volume may become a new marker of depressive state.

Therefore, we investigated the amygdala subregional volume in patients with a first depressive episode and in healthy subjects using image analysis that can measure the amygdala subregional volume.

## Materials and Methods

### Participants

The patients with MD were recruited from the University hospital of University of Occupational and Environmental Health, Japan. All patients were diagnosed by using the full Structured Clinical Interview from the Diagnostic and Statistical Manual for Mental Disorders-5 (American Psychiatric Association, 2013; Cía, 2013). The severity of the depressive state was evaluated using the 17-item Hamilton Rating Scale for Depression (HAMD) (Hamilton, 1960). Patients who met the following criteria were enrolled in the study: (a) a diagnosis of MD, (b) a HAMD score of ≥ 14, (c) first episode and drug-naïveté MD. Thus, a total of 76 first-episode and drug-naïve patients with MD were included. Seventy-seven control participants were also recruited using the Structured Clinical Interview for DSM-5. None of them were applicable for any psychiatric diseases and had a history of serious medical and neurological diseases or a family history of major psychiatric or neurological diseases among their first-degree relatives. Details for the participants enrolled the present study were described in Table 1. Information for smoking status, and Brinkman index were, however, lacked over half of participants. All participants were not met with abuse and dependence with alcohol and substance use.

**TABLE 1:** Background factors in MD patients and healthy control.

	MD patients (*n* = 76)	Healthy control (*n* = 77)	*p*-value
Male/Female	33/43	51/26	<0.01*
Age (year)	53.8 ± 17.0	35.4 ± 12.2	<0.01*
Dominant hand (right/left)	33/2 (unknown data = 41)	50/0 (unknown data = 27)	
Period of education (year)	13.4 ± 2.5 (unknown data = 46)	16.6 ± 2.8 (unknown data = 22)	
Smoking (Yes/No)	16/14 (unknown data = 46)	23/26 (unknown data = 28)	
Brinkman index	330.3 ± 438.1 (unknown data = 46)	179.2 ± 271.8 (unknown data = 44)	
Estimated total intracranial volume	1,521,291 ± 174,390	1,589,616 ± 135,439	<0.01*
HAMD total score (0–52)	22.4 ± 6.30	−	
HAMD subscale score			
Core (0–22)	10.2 ± 3.60	−	
Sleep (0–4)	2.43 ± 1.14	−	
Activity (0–8)	3.82 ± 1.51	−	
Psychic (0–8)	2.78 ± 1.28	−	
Somatic anxiety (0–6)	2.71 ± 1.20	−	

*Statistically Significant (i.e., p-values less than 0.05) is expressed asterisk.*

The subjects in the present study overlapped with those in our published study (Kakeda et al., 2020; Nguyen et al., 2020; Watanabe et al., 2020), however, no study analyzed the amygdala. The study protocol was approved by the Ethics Committee of the University of Occupational and Environmental Health, Japan. Written informed consent was obtained from all patients who participated in this study, which was conducted in accordance with the Declaration of Helsinki.

### MRI Acquisition

MRI data were obtained on a 3T MR system (Signa EXCITE 3T; GE Healthcare, Waukesha, WI) with an 8-channel brain phased-array coil. Images were acquired by three-dimensional fast-spoiled gradient recalled acquisition (3D-FSPGR). The acquisition parameters were: repetition time/echo time, 10/4.1 ms; flip angle, 10°; field of view, 24 cm; and resolution, 0.9 × 0.9 × 1.2 mm. All images were corrected for image distortion due to gradient non-linearity using the “Grad Warp” software program (Jovicich et al., 2006).

### Amygdala Subregion and Hippocampal Subfield Volume

FreeSurfer v7.1.1^1^ (Fischl, 2012) was used for evaluation of Amygdala subregion volumes. This amygdala subregion segmentation technique based on a prior probabilistic atlas and the Bayesian modeling approach is fully automatic (Saygin et al., 2017). Bilateral Amygdala were generated in each subject for the basal, lateral, accessory basal, central, medial, cortical, and paralaminar nuclei as well as the corticoamygdaloid transition area, anterior amygdaloid area, and the whole amygdala. Left and right substructures were analyzed separately. In addition, we simultaneously calculated the hippocampal subfields volume. The hippocampus was generated for the hippocampal tail, subiculum-body, cornus ammonis (CA)1-body, subiculum-head, hippocampal (HP)-fissure, presubiculum-head, CA1-head, presubiculum-body, parasubiculum, molecular-layer-HP-head, molecular-layer-HP-body, granule cell (GC)- molecular layer (ML)- dentate gyrus (DG)-head, CA3-body, GC-ML-DG-body, CA4-head, CA4-body, fimbria, CA3-head, hippocampus-amygdala-transition-area (HATA), Whole_hippocampal_body, Whole_hippocampal_head. Further, the estimated intracranial volume was also calculated using the “aseg segmentation.”

### Assessment of Depressive State

Depression was assessed using the 17-item Hamilton Depression Rating Scale (HAMD). To elucidate the features of depressive symptoms, we classified the HAMD into 5 subscales as HAMD core (items 1, 2, 7, 8, 10, and 13), HAMD sleep (items 4, 6), HAMD activity (items 7, 8), HAMD psychic (items 9, 10), and HAMD somatic anxiety (items 11, 13) (Serretti et al., 1999).

### Statistical Analysis

Covariate-adjusted linear regression was performed to compare amygdala subregion volumes between depressed and normal groups, and to examine the association between amygdala subregion volumes and the HAMD total and the HAMD subscale scores as measures of depression severity. Covariates were adjusted for age, gender, and estimated total intracranial volume. *p*-values less than 0.05 were considered statistically significant. All statistical analyses were performed using EZR software version 4.0.2, which was used to calculate R.

## Results

Analyses were conducted in 76 patients with first-episode depression and 77 healthy subjects. The background factors are shown in Table 1.

### Amygdala Subregion Analysis

**FIGURE 1:**
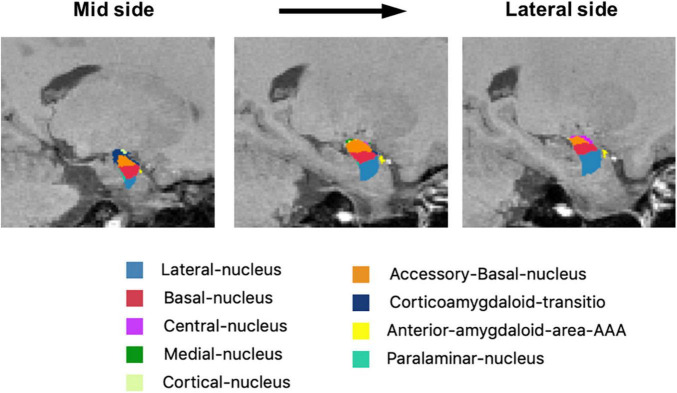
Representative images of the amygdala subregions. The mask of each region overlaid on sagittal T1-weighted images from mid side to lateral side.

**FIGURE 2:**
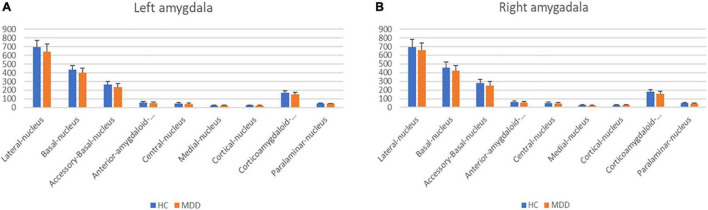
Subregion volumes in amygdala at left **(A)** and right **(B)**. Bar charts show the mean amygdala subregion volumes (mm^3^) of 77 HC (Blue) and 76 MDD (Orange). Vertical bars mean standard deviations. Linear regression analyses showed no significant difference in all subregions between HC and MDD adjusted for total intracranial volume, age, and sex (*p* > 0.05). HC, healthy controls; MDD, Major depressive disorders.

Amygdala subregions were shown in Figure 1. A comparison between the normal group and the depressed group is shown in Tables 2, 3 and Figure 2. There was no difference between the normal and depressed groups. Within the depressed group, the relationship between the amygdala subregion volume and the severity of depression was examined (Table 3A). In the left amygdala, there was no association between amygdala subregion volume and depression severity. In the right amygdala, on the other hand, there was an inverse linear association between the HAMD total and the HAMD core scores and lateral nucleus and anterior-amygdaloid-regions. Furthermore, there was an inverse linear association between the HAMD psychic and lateral nucleus, anterior-amygdaloid-regions, transition, and whole amygdala (Tables 3A,B).

**TABLE 2:** A comparison between the MD patient group and healthy control group in nuclei of amygdala.

	MD patients	Healthy control	*p*-value
**Left**			
Lateral nucleus	645 ± 85.4 mm^3^	693 ± 77.1 mm^3^	0.38
Basal nucleus	399 ± 56.2 mm^3^	437 ± 47.3 mm^3^	0.35
Accessory basal nucleus	236 ± 39.1 mm^3^	267 ± 31.7 mm^3^	0.23
Anterior amygdaloid region	52.9 ± 10.3 mm^3^	59.6 ± 7.60 mm^3^	0.31
Central nucleus	42.5 ± 9.80 mm^3^	48.1 ± 8.80 mm^3^	0.38
Medial nucleus	19.6 ± 5.70 mm^3^	22.9 ± 5.60 mm^3^	0.30
Cortical nucleus	23.1 ± 4.60 mm^3^	26.6 ± 4.20 mm^3^	0.18
Corticoamygdaloid transition	151.9 ± 24.5 mm^3^	171.6 ± 20.3 mm^3^	0.48
Paralaminar nucleus	43.0 ± 5.70 mm^3^	46.0 ± 5.00 mm^3^	0.68
Whole amygdala	1,613 ± 220 mm^3^	1,771 ± 182 mm^3^	0.26
**Right**			
Lateral nucleus	656 ± 86.0 mm^3^	693 ± 87.3 mm^3^	0.48
Basal nucleus	420 ± 58.4 mm^3^	459 ± 64.9 mm^3^	0.89
Accessory basal nucleus	254 ± 42.0 mm^3^	281 ± 39.4 mm^3^	0.84
Anterior amygdaloid region	56.2 ± 8.90 mm^3^	64.0 ± 11.6 mm^3^	0.46
Central nucleus	46.0 ± 9.80 mm^3^	49.7 ± 10.2 mm^3^	0.61
Medial nucleus	21.7 ± 6.50 mm^3^	23.6 ± 6.40 mm^3^	0.47
Cortical nucleus	25.1 ± 5.10 mm^3^	27.7 ± 4.40 mm^3^	0.46
Corticoamygdaloid transition	159 ± 26.3 mm^3^	180 ± 24.4 mm^3^	0.60
Paralaminar nucleus	44.0 ± 6.10 mm^3^	47.1 ± 6.60 mm^3^	0.93
Whole amygdala	1,682 ± 226 mm^3^	1,825 ± 232 mm^3^	0.81

*The volumes were given under: mean ± S.D, Age, sex and total intracranial volume are covariates in analyses.*

**TABLE 3A:** The HAMD scores and amygdala subregions.

	*HAMD total score*	HAMD subscale score				
		Core	Sleep	Activity	Psychic	Somatic anxiety
**Left**						
Lateral nucleus	0.16	0.33	0.42	0.78	0.05	0.40
Basal nucleus	0.82	0.57	0.66	0.66	0.30	0.64
Accessory basal nucleus	0.97	0.85	0.92	0.94	0.96	0.63
Anterior amygdaloid region	0.36	0.43	0.23	0.21	0.53	0.83
Central nucleus	0.95	0.75	0.39	0.93	0.32	0.27
Medial nucleus	0.56	0.98	0.12	0.97	0.50	0.21
Cortical nucleus	0.54	0.43	0.55	0.39	0.86	0.24
Corticoamygdaloid transition	0.92	0.87	0.35	0.61	0.69	0.99
Paralaminar nucleus	0.49	0.26	0.27	0.25	0.09	0.88
Whole amygdala	0.55	0.55	0.91	0.79	0.26	0.95
**Right**						
Lateral nucleus	0.010*	0.020*	0.090	0.27	0.0060*	0.27
Basal nucleus	0.31	0.31	0.56	0.59	0.080	0.62
Accessory basal nucleus	0.58	0.72	0.87	0.71	0.17	0.53
Anterior amygdaloid region	0.030*	0.020*	0.67	0.30	0.040*	0.070
Central nucleus	0.97	0.78	0.97	0.69	0.38	0.39
Medial nucleus	0.45	0.46	0.90	0.22	0.42	0.030*
Cortical nucleus	0.62	0.44	0.96	0.09	0.35	0.35
Corticoamygdaloid transition	0.36	0.31	0.28	0.64	0.040*	0.10
Paralaminar nucleus	0.35	0.36	0.63	0.57	0.050	0.74
Whole amygdala	0.13	0.16	0.47	0.62	0.020*	0.43

*All data are p-values. Age, sex, and total intracranial volume are covariates in analyses. Statistically Significant (i.e., p-value less than 0.05) is expressed asterisk.*

**TABLE 3B:** The HAMD scores and amygdala subregions (Heat Map).

	HAMD total score	HAMD subscale score				
		Core	Sleep	Activity	Psychic	Somatic anxiety

	**B (95%CI)** ***p*-value**			**B (95%CI) *p*-value**		
**Right**						
Lateral nucleus	−2.62 (−4.70, −0.54) (*p* = 0.010*)	−4.32 (−8.06, −0.57) (*p* = 0.020*)	−10.2 (−22.0, 1.70) (*p* = 0.090)	−4.95 (13.9, 4.04) (*p* = 0.27)	−14.3 (−24.5, −4.07) (*p* = 0.0060*)	−6.38 (17.9, 5.18) (*p* = 0.27)
Anterior amygdaloid region	−0.27 (−0.51, −0.02) (*p* = 0.030*)	−0.48 (−0.91, 0.05) (*p* = 0.020*)	−0.29 (−1.68, 1.10) (*p* = 0.67)	−0.53 (−1.56, 0.50) (*p* = 0.31)	−1.24 (−2.44, −0.04) (*p* = 0.040*)	−1.21 (0.09, 2.40) (*p* = 0.030*)
Medial nucleus	0.08 (−1.06, 0.40) (*p* = 0.36)	0.14 (−0.25, 0.54) (*p* = 0.46)	−0.07 (−1.31, 1.16) (*p* = 0.91)	0.56 (−0.36, 1.48) (*p* = 0.22)	−0.44 (−1.54, 0.66) (*p* = 0.42)	1.25 (0.09, 2.40) (*p* = 0.030*)
Corticoamygdaloid transition	−0.33 (−1.06, 0.40) (*p* = 0.36)	−0.66 (−1.95, 0.63) (*p* = 0.31)	2.2 (−1.83, 6.23) (*p* = 0.28)	−0.70 (−3.74, 2.34) (*p* = 0.064)	−3.66 (−7.18, −0.14) (*p* = 0.040*)	−3.13 (−6.97, 0.72) (*p* = 0.11)
Whole amygdala	−4.40 (−10.22, 1.42) (*p* = 0.13)	0−7.41 (−17.8, 3.00) (*p* = 0.16)	1−11.9 (−44.7, 20.9) (*p* = 0.47)	2−6.08 (−30.7, 18.5) (*p* = 0.62)	3−33.13 (−61.4, −4.88) (*p* = 0.020*)	4−12.4 (−44.0, 19.2) (*p* = 0.43)

*B is the partial regression coefficient. CI is the confidence interval. Statistically Significant (i. e. p-values less than 0.05) is expressed asterisk. Partial regression coefficient is represented in a heat map. Negative values are shown in orange, positive values are shown in blue, and the strength of the number is expressed in terms of concentration (color scale wis set up minimum −30 to maximum 30).*

### Hippocampal Subfields Analysis

**FIGURE 3:**
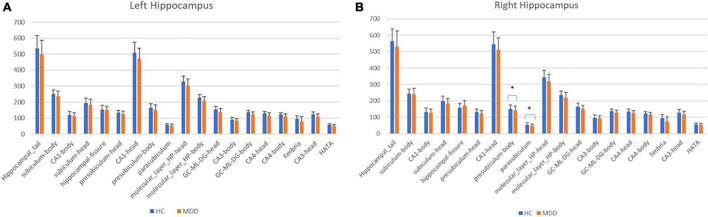
Subfield volumes in hippocampus at left **(A)** and right **(B)**. Bar charts show the mean hippocampal subfields volumes (mm^3^) of 77 HC (Blue) and 76 MDD (Orange). Vertical bars mean standard deviations. Asterisks show statistically significance (*p* < 0.05). Linear regression analyses showed significant difference between HC and MDD only in the right presubiculum-body and right parasubiculum adjusted for total intracranial volume, age, and sex (*p* < 0.01 and *p* = 0.04, respectively). There was no significance in other subfields (*p* > 0.05). HC, healthy controls; MDD, Major depressive disorders.

Table 4 and Figure 3 also showed a comparison between the normal group and the depressed group in the hippocampal subfields. The depressed group showed significantly decreased volumes of the right subiculum-body and parasubiculum (*p* < 0.01, 0.04, respectively). In the analysis with depression severity (Tables 5A,B), there were some significant inverse linear associations; (1) the HAMD total and the right HATA and left CA3-head, (2) The HAMD core and the right HATA, left CA3-head, and left GC-ML-DG-head, (3) The HAMD activity and the right HATA and left CA3-head, (4) The HAMD psychic and the right HATA. On the other hand, the HAMD sleep showed positive correlation with the left subiculum-head, parasubiculum-head, and HATA.

**TABLE 4:** A comparison between the MD patient group and healthy control group in Hippocampal subfields.

	MD patients	Healthy control	*p*-value
**Left**			
Hippocampal_tail	498 ± 88.4 mm^3^	533 ± 83.8 mm^3^	0.61
Subiculum-body	237 ± 33.4 mm^3^	248 ± 29.0 mm^3^	0.24
CA1-body	111 ± 22.6 mm^3^	119 ± 23.0 mm^3^	0.88
Subiculum-head	183 ± 33.5 mm^3^	192 ± 32.1 mm^3^	0.34
Hippocampal-fissure	152 ± 21.5 mm^3^	152 ± 29.5 mm^3^	0.63
Presubiculum-head	125 ± 18.6 mm^3^	133 ± 16.6 mm^3^	0.90
CA1-head	471 ± 67.0 mm^3^	506 ± 69.3 mm^3^	0.89
Presubiculum-body	151 ± 31.7 mm^3^	161 ± 28.5 mm^3^	0.59
Parasubiculum	50.2 ± 11.0 mm^3^	55.4 ± 10.3 mm^3^	0.12
Molecular-layer-HP-head	304 ± 40.6 mm^3^	327 ± 37.2 mm^3^	0.79
Molecular-layer-HP-body	207 ± 27.4 mm^3^	225 ± 22.3 mm^3^	0.42
GC-ML-DG-head	140 ± 20.9 mm^3^	153 ± 18.9 mm^3^	0.31
CA3-body	83.3 ± 14.1 mm^3^	87.7 ± 15.9 mm^3^	0.62
GC-ML-DG-body	123 ± 15.2 mm^3^	134 ± 13.4 mm^3^	0.10
CA4-head	117 ± 16.2 mm^3^	126 ± 14.9 mm^3^	0.46
CA4-body	112 ± 13.6 mm^3^	120 ± 13.1 mm^3^	0.23
Fimbria	80.3 ± 28.2 mm^3^	94.0 ± 20.1 mm^3^	0.10
CA3-head	109 ± 18.2 mm^3^	119 ± 18.8 mm^3^	0.30
HATA	50.1 ± 9.28 mm^3^	55.7 ± 8.51 mm^3^	0.25
Whole_hippocampal_body	1,104 ± 133 mm^3^	1,189 ± 97.6 mm^3^	0.61
Whole_hippocampal_head	1,549 ± 195 mm^3^	1,667 ± 182 mm^3^	0.70
Whole_hippocampus	3,151 ± 370 mm^3^	3,388 ± 293 mm^3^	0.81
**Right**			
Hippocampal_tail	530 ± 95.9 mm^3^	565 ± 74.8 mm^3^	0.53
Subiculum-body	240 ± 36.2 mm^3^	245 ± 26.4 mm^3^	<0.01*
CA1-body	127 ± 23.5 mm^3^	131 ± 25.4 mm^3^	0.10
Subiculum-head	183 ± 31.8 mm^3^	197 ± 32.2 mm^3^	0.64
Hippocampal-fissure	170 ± 30.8 mm^3^	158 ± 25.9 mm^3^	0.06
Presubiculum-head	122 ± 19.3 mm^3^	133 ± 18.1 mm^3^	0.10
CA1-head	512 ± 72.1 mm^3^	545 ± 74.5 mm^3^	0.73
Presubiculum-body	141 ± 26.3 mm^3^	151 ± 23.6 mm^3^	0.39
Parasubiculum	47.2 ± 11.9 mm^3^	54.5 ± 12.6 mm^3^	0.04*
Molecular_layer_HP-head	319 ± 42.9 mm^3^	343 ± 42.1 mm^3^	0.83
Molecular_layer_HP-body	219 ± 31.4 mm^3^	235 ± 25.8 mm^3^	0.41
GC-ML-DG-head	150 ± 23.0 mm^3^	165 ± 21.2 mm^3^	0.61
CA3-body	94.8 ± 15.2 mm^3^	97.3 ± 16.3 mm^3^	0.11
GC-ML-DG-body	127 ± 16.6 mm^3^	137 ± 13.4 mm^3^	0.42
CA4-head	125 ± 17.1 mm^3^	135 ± 16.2 mm^3^	0.69
CA4-body	116 ± 15.0 mm^3^	122 ± 12.2 mm^3^	0.81
Fimbria	72.8 ± 30.6 mm^3^	94.8 ± 22.0 mm^3^	0.15
CA3-head	117 ± 20.4 mm^3^	128 ± 18.8 mm^3^	0.78
HATA	53.4 ± 10.0 mm^3^	56.9 ± 8.57 mm^3^	0.70
Whole-hippocampal-body	1,138 ± 149 mm^3^	1,214 ± 113 mm^3^	0.42
Whole-hippocampal-head	1,631 ± 207 mm^3^	1,758 ± 208 mm^3^	0.68
Whole-hippocampus	3,299 ± 410 mm^3^	3,537 ± 350 mm^3^	0.84

*The volumes were normalized and given under: mean ± S.D, Age, sex, and total intracranial volume are covariates in analyses. Statistically Significant (i.e., p-values less than 0.05) is expressed asterisk.*

**TABLE 5A:** The HAMD scores and hippocampus subfields.

	HAMD total score	HAMD subscale score				
		Core	Sleep	Activity	Psychic	Somatic anxiety
**Left**						
Hippocampal tail	0.99	0.73	0.48	0.66	0.67	0.34
Subiculum body	0.94	0.92	0.71	0.81	0.56	0.82
CA1 body	0.95	0.59	0.84	0.75	0.70	0.64
Subiculum head	0.28	0.78	0.012*	0.75	0.86	0.55
Hippocampal fissure	0.44	0.52	0.98	0.22	0.83	0.71
Presubiculum head	0.47	0.90	0.0030*	0.69	0.50	0.72
CA1 head	0.41	0.84	0.37	0.66	0.82	0.91
Presubiculum body	0.28	0.37	0.21	0.69	0.50	0.31
Parasubiculum	0.86	0.88	0.24	0.55	0.53	0.90
Molecular layer HP head	0.60	0.98	0.19	0.90	0.76	0.63
Molecular layer HP body	0.71	0.97	0.48	0.83	0.46	0.95
GC ML DG head	0.07	0.042*	0.75	0.11	0.30	0.20
CA3 body	0.58	0.43	0.77	0.52	0.85	0.21
GC ML DG body	0.66	0.79	0.90	0.86	0.98	0.92
CA4 head	0.08	0.07	0.48	0.16	0.34	0.29
CA4 body	0.63	0.84	0.96	0.89	0.98	0.60
Fimbria	0.07	0.10	0.84	0.31	0.29	0.15
CA3 head	0.015*	0.007*	0.44	0.025*	0.15	0.15
HATA	0.38	0.16	0.025*	0.09	0.91	0.31
Whole hippocampal body	0.93	0.73	0.46	0.73	0.92	0.71
Whole hippocampal head	0.94	0.54	0.13	0.63	0.98	0.51
Whole hippocampus	0.999	0.58	0.20	0.61	0.92	0.44
**Right**						
Hippocampal tail	0.55	0.52	0.67	0.74	0.19	0.65
Subiculum body	0.95	0.92	0.97	0.52	0.43	0.68
CA1 body	0.84	0.89	0.79	0.50	0.53	0.73
Subiculum head	0.76	0.97	0.87	0.62	0.93	0.95
Hippocampal fissure	0.98	0.61	0.08	0.50	0.76	0.74
Presubiculum head	0.95	0.47	0.24	0.65	0.99	0.46
CA1 head	0.44	0.67	0.83	0.45	0.93	0.63
Presubiculum body	0.91	0.60	0.68	0.67	0.74	0.21
Parasubiculum	0.59	0.20	0.48	0.32	0.28	0.40
Molecular layer HP head	0.71	0.98	0.83	0.64	0.94	0.97
Molecular layer HP body	0.97	0.82	0.84	0.77	0.60	0.93
GC ML DG head	0.30	0.23	0.72	0.72	0.29	0.13
CA3 body	0.70	0.67	0.91	0.88	0.32	0.67
GC ML DG body	0.43	0.49	0.70	0.52	0.43	0.34
CA4 head	0.22	0.20	0.90	0.61	0.15	0.15
CA4 body	0.32	0.50	0.63	0.57	0.39	0.19
Fimbria	0.12	0.47	0.75	0.77	0.59	0.12
CA3 head	0.46	0.23	0.23	0.46	0.29	0.21
HATA	0.030*	0.0052*	0.31	0.0066*	0.19	0.0087*
Whole hippocampal body	0.61	0.60	0.91	0.94	0.43	0.72
Whole hippocampal head	0.98	0.61	0.73	0.96	0.63	0.63
Whole hippocampus	0.72	0.53	0.74	0.97	0.38	0.62

*All data are p-values. Age, sex and total intracranial volume are covariates in analyses. Statistically Significant (i.e., p-value less than 0.05) is expressed asterisk.*

**TABLE 5B:** The HAMD scores and hippocampus subfields (Heat Map).

	HAMD total score	HAMD subscale score				
		Core	Sleep	Activity	Psychic	Somatic anxiety

	B (95%CI) *p*-value	B (95%CI) *p*-value				
**Right**						
HATA	−0.36 (−0.68, −0.04) (*p* = 0.030*)	−0.81 (−1.37, −0.25) (*p* = 0.005*)	0.94 (−0.89, 2.78) (*p* = 0.31)	−1.84 (−3.16, −0.53) (*p* = 0.007*)	−1.08 (−2.71, 0.55) (*p* = 0.19)	−2.30 (−3.99, −0.60) (*p* = 0.009*)
**Left**						
Subiculum head	0.58 (−0.49, 1.64) (*p* = 0.28)	0.27 (−1.64, 2.18) (*p* = 0.78)	7.40 (1.69, 13.1) (*p* = 0.012*)	0.72 (−3.75, 5.18) (*p* = 0.75)	0.46 (−4.86, 5.77) (*p* = 0.86)	−1.73 (−7.45, 4.01) (*p* = 0.55)
Presubiculum head	0.24 (−0.42, 0.89) (*p* = 0.47)	−0.07 (−1.25, 1.10) (*p* = 0.90)	5.28 (1.85, 8.71) (*p* = 0.003*)	0−0.56 (−3.29, 2.18) (*p* = 0.69)	1.10 (−2.14, 4.35) (*p* = 0.50)	−0.64 (−4.15, 2.88) (*p* = 0.72)
GC ML DG head	−0.52 (−1.06, 0.04) (*p* = 0.07)	−1.02 (−2.0, −0.04) (*p* = 0.04*)	−0.51 (−3.65, 2.63) (*p* = 0.75)	−1.88 (−4.19, 0.44) (*p* = 0.11)	−1.46 (−4.24, 1.32) (*p* = 0.30)	−1.93 (−4.93, 1.07) (*p* = 0.20)
CA3 head	−0.68 (−1.22, −0.13) (*p* = 0.015*)	−1.33 (−2.30, −0.37) (*p* = 0.007*)	−1.24 (−4.39, 1.91) (*p* = 0.44)	−2.62 (−4.91, −0.34) (*p* = 0.025*)	−2.03 (−4.80, 0.75) (*p* = 0.15)	−2.17 (−5.17, 0.84) (*p* = 0.15)
HATA	−0.13 (−0.42, 0.16) (*p* = 0.37)	−0.37 (−0.89, 0.15) (*p* = 0.16)	1.84 (0.24, 3.44) (*p* = 0.024*)	−1.03 (−2.25, 0.18) (*p* = 0.09)	0.08 (−1.39, 1.55) (*p* = 0.91)	−0.81 (−2.39, 0.77) (*p* = 0.31)

*B is the partial regression coefficient. CI is the confidence interval. Statistically Significant (i. e. p-values less than 0.05) is expressed asterisk. Partial regression coefficient is represented in a heat map. Negative values are shown in orange, positive values are shown in blue, and the strength of the number is expressed in terms of concentration (color scale is set up minimum −10 to maximum 10).*

## Discussion

In the present study, no differences in amygdala subregion volumes were observed between the healthy and depressed groups. However, examination of the relationship between the amygdala subregion volume and the severity of depression in the depressed group found that the greater the severity of the whole depressive symptom and the core symptoms of depression, the smaller the lateral nucleus and anterior-amygdaloid-regions in the right amygdala, and the greater the severity of anxiety and agitation, the smaller the lateral nucleus, anterior-amygdaloid-regions, transition, and whole amygdala. The finding that the right amygdala is associated with the severity of depression is consistent with a previous report (Gaffrey et al., 2011). In our results, the lateral nucleus and anterior-amygdaloid-regions were strongly related to the HAMD total score in depression. In terms of neural circuits involved in fear conditioning, both conditioned and unconditioned stimuli are sent to the lateral nucleus of the amygdala, which is thought to be responsible for the association between conditioned and unconditioned stimuli exploring the molecular mechanisms of learning and memory (LeDoux, 2000, 2007; Johansen et al., 2011). Although three pathways from the lateral nucleus to the medial central nucleus have been postulated (Pape and Pare, 2010), they are important relay sites for both stimulus input and output. Therefore, these regions may be sensitive to stressful stimuli. Li et al. (2021) investigated structural and functional alterations of amygdala subregions among with anxious major depressive disorder patients, non-anxious major depressive disorder patients, and healthy controls using structural MRI and resting-state functional MRI. The authors fond that there were no significant differences in the gray matter volume among the three groups. Kim et al. (2021) reported that volumes of right whole amygdala, right lateral nucleus, right anterior amygdala were lower in patients with major depressive disorder than in healthy controls. The authors also found that no significant associations between subregion volumes of amygdala and antidepressant use, illness duration, or depressive severity. The result of our study that no differences in amygdala subregions volumes between the healthy controls and MD patients was in accordance with the result of Li et al. (2021). The discrepancy existed regarding the associations of the amygdala subregions and the HAMD scores in our results and the results of Kim et al. (2021). The reasons for the discrepancy remain unknown. It has been reported that amygdala volume was negatively related to illness duration, suggesting volume loss with disease progression (Zavorotnyy et al., 2018). A meta-analysis (Hamilton et al., 2008) found that medicated amygdala volume was significantly increased in medicated depressive patients relative to healthy persons, whereas, unmedicated depressed patients showed a reliable decrease in amygdala volume. Lithium, a mood stabilizer increased amygdala volume in depressed patients (Savitz et al., 2010). A significant inverse correlation between amygdala volume and the number of preceding depressive episodes (Kronenberg et al., 2009). Moreover, psychotic but not non-psychotic depression was associated with reduced amygdala volume (Keller et al., 2008). From these findings into account, several factors including duration of illness, medications such as antidepressants and lithium, depressive phenotype, i.e., anxious depression, non-anxious depression, psychotic depression, or non-psychotic depression might influence the results. Also, the results of amygdala volume in MD are controversial because of the heterogeneity of the disease. It is well known projection from basolateral amygdala to central nucleus of the amygdala as critical circuit elements for anxiety in the mammalian brain (Tye et al., 2011). The circuitry of the extended amygdala, including the central and basolateral nuclei of the amygdala and the bed nucleus of the stria terminalis is associated with anxiety (Weis et al., 2019). Recent study reported that projection from basolateral amygdala to olfactory tubercle or central nucleus of the amygdala are associated with positive- or negative-valence of emotion (Zhang et al., 2021).

Additionally, focusing on the HAMD psychic (items 9 and 10) scores rather than the HAMD total scores in depression, the increased number of amygdala subregions suggests that agitation and anxiety psychic, that is, agitation and mental anxiety, may have a profound effect on the neural circuits in the brain, especially in the amygdala. Previous studies have suggested that the amygdala plays a central role in anxiety disorders, and there has been a report that local administration of SSRIs or serotonin1A receptor agonists around the lateral nucleus of the bilateral amygdala produced anxiolytic effects (Izumi et al., 2008). It has also been reported that polymorphisms in the serotonin transporter gene are involved in the sensitivity of the amygdala, and in individuals with the S allele, there was a correlation with activation of the right amygdala. This result indicates that the function of the serotonin transporter is involved in the formation of negative emotions (Heinz et al., 2005). In a comparison of children and adults with depression and anxiety disorders with normal controls, it was reported that children and adults with anxiety disorders showed higher activity in the right amygdala in response to phobic symptoms (Thomas et al., 2001). This suggests that increased activity in the networks controlling anxiety, fear, and avoidance may already be present in childhood. As mentioned above, there are many previous studies that have shown a relationship between agitation and anxiety and the right amygdala (Gaffrey et al., 2011), and the present results are consistent with these studies. It has been reported that depression can be classified into two types: one with anxiety symptoms in the foreground and the other without anxiety symptoms, and that those with anxiety symptoms do not respond well to antidepressants. MD may be classified into two subtypes: one in which the amygdala plays a major role, and the other in which it does not. Furthermore, subtyping of MD using the amygdala as a state marker may lead to the prediction and prognosis of depression, as well as to the individualization of treatment strategies for drug therapy.

Regarding the hippocampal subfields analysis, the right subiculum body and parasubiculum volume were significantly decreased in depressed group. In previous studies, the highest changes in the subiculum and CA1, followed by the CA2-3, were reported not only in first-episode MD (Isikli et al., 2013), but also in elderly depression (Ballmaier et al., 2008). The subiculum, in which the anterior region, is associated with emotional functioning (Moser and Moser, 1998), plays an important role in the pathophysiology of MD. On the other hand, there was no significant volume reduction in the amygdala subregions. The decreased volumes of both hippocampus and amygdala in first episode MD have been well reported (van Erp et al., 2016). However, in the resent-onset MD, the enlarged amygdala volume and reduced hippocampal volume can exist (Lange and Irle, 2004). The decreased hippocampal subfield volume and little change amygdala subregion volume may be the process which the amygdala changes from enlarged to reduced volume. In the analysis with severity of depression, inversed relationships were shown mainly in the right HATA and left CA3-head. The HATA is a part of the hippocampus—amygdala circuitry, more specifically it represents one of the targets of the hippocampal- amygdala projection (Fudge et al., 2012; p. 6). However, it has not been well investigated in MD. The CA3 appear to be rich in glucocorticoid receptors and vulnerable to excess cortisol (Magariños et al., 2011). Therefore, the CA3 was considered to be associated with brain damage in MD. Interestingly, the hippocampal subfields that did not show a significant volume reduction correlated with the severity of depression, similar to the amygdala subregion analysis. One possible explanation is that some MD patients showed the slight volume reduction in the CA3 or HATA and more severe symptoms, but the mean volume of CA3 or HATA was not significantly decreased in all MD patients. Regarding the HAMD sleep, the left subiculum-head, parasubiculum-head, and HATA were positively correlated, not negatively correlated. Future studies are needed to elucidate the relationship between the hippocampal subfields and depression severity.

## Limitations

There are several limitations to this study. First, most of the healthy subjects were related to our university, and many of them were doctors, nurses, and other medical professionals. Therefore, the generalizability of the results is uncertain. In addition, the number of cases is limited because the study was conducted on first-episode depression patients without pharmacotherapy.

Second, the severity of depression was determined only by the HAMD, assessing other rating scale such as Montgomery-Asberg Depression Rating Scale might reinforce the accuracy in the severity of depression (Montgomery and Asberg, 1979).

Third, because this was a cross-sectional study, the temporal relationship between depressive symptoms and amygdala subregion volume is unknown.

Fourth, there was no difference between the healthy and depressed groups, which raises questions about the relationship between depression severity and amygdala subregion volume. It is possible that some subjects in the normal group were in a depressive state but did not seek medical attention.

## Conclusion

The present study suggests that the subregional volume of the right amygdala is associated with total severity of depression and the severity of items such as anxiety/agitation. Since there are only a few reports on depression and the amygdala, data accumulation is necessary for further verification.

## Data Availability Statement

The original contributions presented in the study are included in the article/supplementary material, further inquiries can be directed to the corresponding author/s.

## Ethics Statement

The studies involving human participants were reviewed and approved by the Ethics Committee of the University of Occupational and Environmental Health, Japan. The patients/participants provided their written informed consent to participate in this study.

## Author Contributions

HT, KW, SK, and RY conceived and designed the experiments. HT, KW, SK, AI, NO, and RY performed the experiments. HT, KW, and NO analyzed the data. HT, KW, and RY composed the manuscript. HT, KW, AI, SK, and RY provided expertise and edited the manuscript. All authors read the manuscript and are solely and jointly responsible for its content.

## Conflict of Interest

The authors declare that the research was conducted in the absence of any commercial or financial relationships that could be construed as a potential conflict of interest.

## Publisher’s Note

All claims expressed in this article are solely those of the authors and do not necessarily represent those of their affiliated organizations, or those of the publisher, the editors and the reviewers. Any product that may be evaluated in this article, or claim that may be made by its manufacturer, is not guaranteed or endorsed by the publisher.

## Footnotes

1
http://surfer.nmr.mgh.harvard.edu/
